# HER2 Exon 20 Insertion Mutations in Lung Adenocarcinoma: Case Series and Response to Pyrotinib

**DOI:** 10.3389/fonc.2020.01162

**Published:** 2020-07-31

**Authors:** Xinyong Zhang, Jialin Lv, Yuhua Wu, Na Qin, Li Ma, Xi Li, Jingying Nong, Hui Zhang, Quan Zhang, Xinjie Yang, Huibo Shi, Jinghui Wang, Shucai Zhang

**Affiliations:** ^1^Department of Medical Oncology, Beijing Tuberculosis and Thoracic Tumor Research Institute, Beijing Chest Hospital, Capital Medical University, Beijing, China; ^2^Organ Transplantation Research Institute, Tongji Hospital, Tongji Medical College, Huazhong University of Science and Technology, Wuhan, China

**Keywords:** lung adenocarcinoma, human epidermal growth factor receptor 2, exon 20, driver oncogenes, pyrotinib

## Abstract

HER2 mutations have emerged as oncogenic driver gene mutations in non-small cell lung cancer (NSCLC), which have not been described in detail like other driver gene mutations. Here, 295 patients with advanced lung adenocarcinoma were retrospectively screened for HER2 mutations using next-generation sequencing (NGS), and the positive cases were validated by Sanger sequencing. We identified five cases with HER2 exon 20 insertions, representing 1.7% of 295 lung adenocarcinomas. Among them, four different subtypes of HER2 exon 20 insertions were identified, including a rare subtype G778_S779insCPG never reported before with a partial response (PR) to pyrotinib and progression-free survival (PFS) of 12.8 months. Our findings reveal that HER2 exon 20 insertion mutations were detected in a small subset of lung adenocarcinomas. Given the different drug sensitivities, determining the mutation subtype by next-generation sequencing at the time of diagnosis might make sense.

## Introduction

Lung cancer is still the leading cause of cancer-related death around the world (1). Research progress in molecular biology in lung cancer has revealed insights into various crucial signal pathways that control cell survival and proliferation and identified many driver gene mutations that are responsible for cancer development and progression. Therapies targeted on some of these driver gene mutations, such as epidermal growth factor receptor (EGFR)-activating mutations (2), anaplastic lymphoma kinase (ALK) rearrangement (3), and ROS1 rearrangement (4), are associated with a higher response rate (RR) and longer progression-free survival (PFS) than chemotherapy in the clinical practice.

Human epidermal growth factor receptor 2 (HER2/ERBB2) is a member of the ERBB receptor tyrosine kinase family. The ERBB2 gene, which encodes for HER2, is a major proliferative driver that stimulates downstream signaling through PI3K-AKT and MEK-ERK pathways (5). HER2 mutations consist of in-frame insertions in exon 20, leading to constitutive activation of the receptor and downstream AKT and MEK pathways (6).

HER2 mutations have emerged as therapeutic targets in non-small cell lung cancers (NSCLC), occurring in 1.7–3.33% (7, 8). The most common mutations are in-frame insertions in exon 20, especially the A775_G776insYVMA insertion/duplication (7–9). HER2 mutations have been previously shown to be mutually exclusive with EGFR and ALK rearrangements (7, 9).

Among reported lung cancer biomarkers, HER2 as a target remains poorly described, partly because of its lower incidence without potent targeted therapies. Although results from early phase II trials of trastuzumab as a treatment for HER2-mutant NSCLC have not shown any advantage for most patients, subsequent clinical trials have reported objective responses to afatinib (10, 11), poziotinib (12, 13), dacomitinib (14), neratinib plus temsirolimus (15), trastuzumab (16), trastuzumab emtansine (T-DM1) (17), and pyrotinib (18) in patients with HER2-mutant lung cancers (8). In general, patients with HER2 mutations are resistant to EGFR tyrosine kinase inhibitors (TKIs), but part of them are sensitive to both HER2 inhibitors and dual EGFR/HER2 inhibitors.

Here, we present five NSCLC cases with HER2 exon 20 insertion mutations to show the different subtypes and the heterogeneity in clinical response. Notably, we identified a patient with a rare subtype of HER2 exon 20 mutation G778_S779insCPG who experienced a partial response (PR) to pyrotinib after failure of first-line chemotherapy and second-line immunotherapy.

## Materials and Methods

We retrospectively collected 295 patients with pathologically confirmed lung adenocarcinoma (stage IIIB/IV) from January 2017 to March 2019 in Beijing Chest Hospital, Capital Medical University, whose tumor tissue samples were screened for HER2 mutations using next-generation sequencing (NGS). Sanger sequencing was performed in positive cases. The clinical data were collected, including age, gender, smoking status, tumor histology, performance status (PS), and the outcomes of anti-cancer therapies if possible. Tumor response to targeted therapy was evaluated 1 month after the therapy and every 2 months according to Response Evaluation Criteria in Solid Tumors (RECIST version 1.1). Adverse events were evaluated according to the National Cancer Institute (USA) Common Terminology Criteria for Adverse Events, version 4.01 (NCI CTCAE v4.01). The study was approved by an institutional review board in Beijing Chest Hospital, Capital Medical University. All patients provided written informed consent.

## Results

### Patient Characteristics

A total of 295 patients were pathologically confirmed with adenocarcinoma in stage III/IV. We identified five patients harboring HER2 exon 20 insertion mutations from 295 lung adenocarcinoma patients with a prevalence of 1.7%. The characteristics of patients with HER2 mutations were shown in Table 1. The median age was 64 years old, ranging from 58 to 66 years old. There were three females who never smoked and two males who were current smokers. All of them were stage IV.

**Table 1:** Characteristics of patients with HER2 mutations (*N* = 5).

**Patient**	**Gender/age**	**Smoking**	**Histological types**	**HER2 mutation subtypes**	**Concurrent mutation**	**Subsequent treatment**
1	M/58	Current	Adenocarcinoma	p.A775_G776insYVMA	HER2 amplification	Chemotherapy
2	F/66	Never	Adenocarcinoma	p.A775_G776insYVMA	No	No
3	M/64	Current	Adenocarcinoma	p.778insGCP	EML4-ALK	Chemotherapy
4	F/65	Never	Adenocarcinoma	p.G778_S779insCPG	No	Pyrotinib
5	F/60	Never	Adenocarcinoma	p.G780_P781dupGSP	No	Pyrotinib

### Subtypes of HER2 Mutation

Among them, four different subtypes of HER2 exon 20 insertions were identified (Table 1). The most common mutation was A775_G776insYVMA, which was found in two of five cases. The other three were detected only once, including p.778insGCP, p.G780_P781dupGSP, and p.G778_S779insCPG. Moreover, two cases carried concurrent mutations. One had ERBB2 amplification together with p.A775_G776insYVMA. The other one had EML4-ALK fusion (EML4 exon13-ALK exon 20) and p.778insGCP concurrently.

### Treatment Outcomes

**Figure 1:**
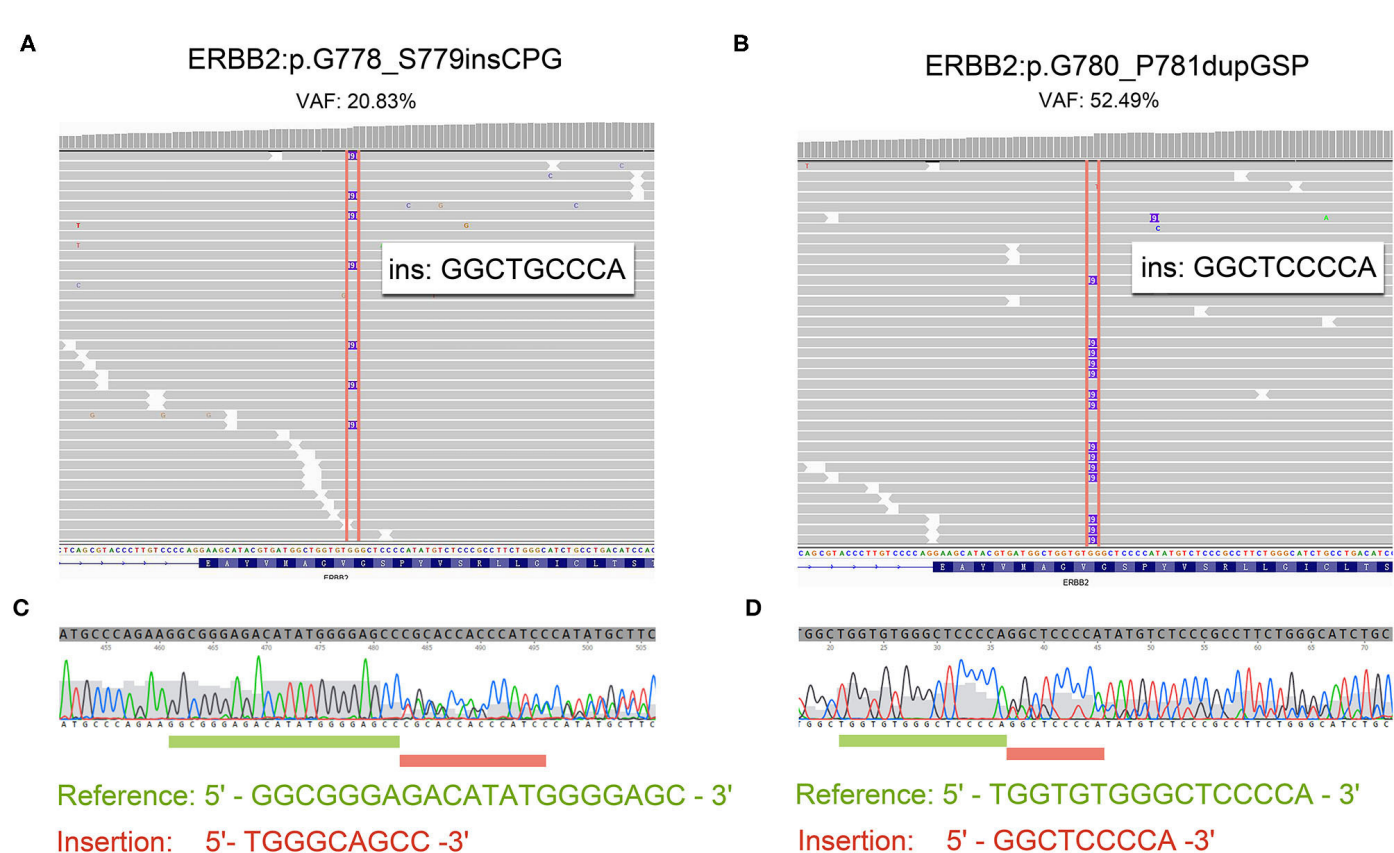
The reads of 20 insertion subtypes identified by NGS and Sanger sequencing. **(A,C)** Patient 4 with G780_P781dupGSP; **(B,D)** Patient 5 with G778_S779insCPG.

**Figure 2:**
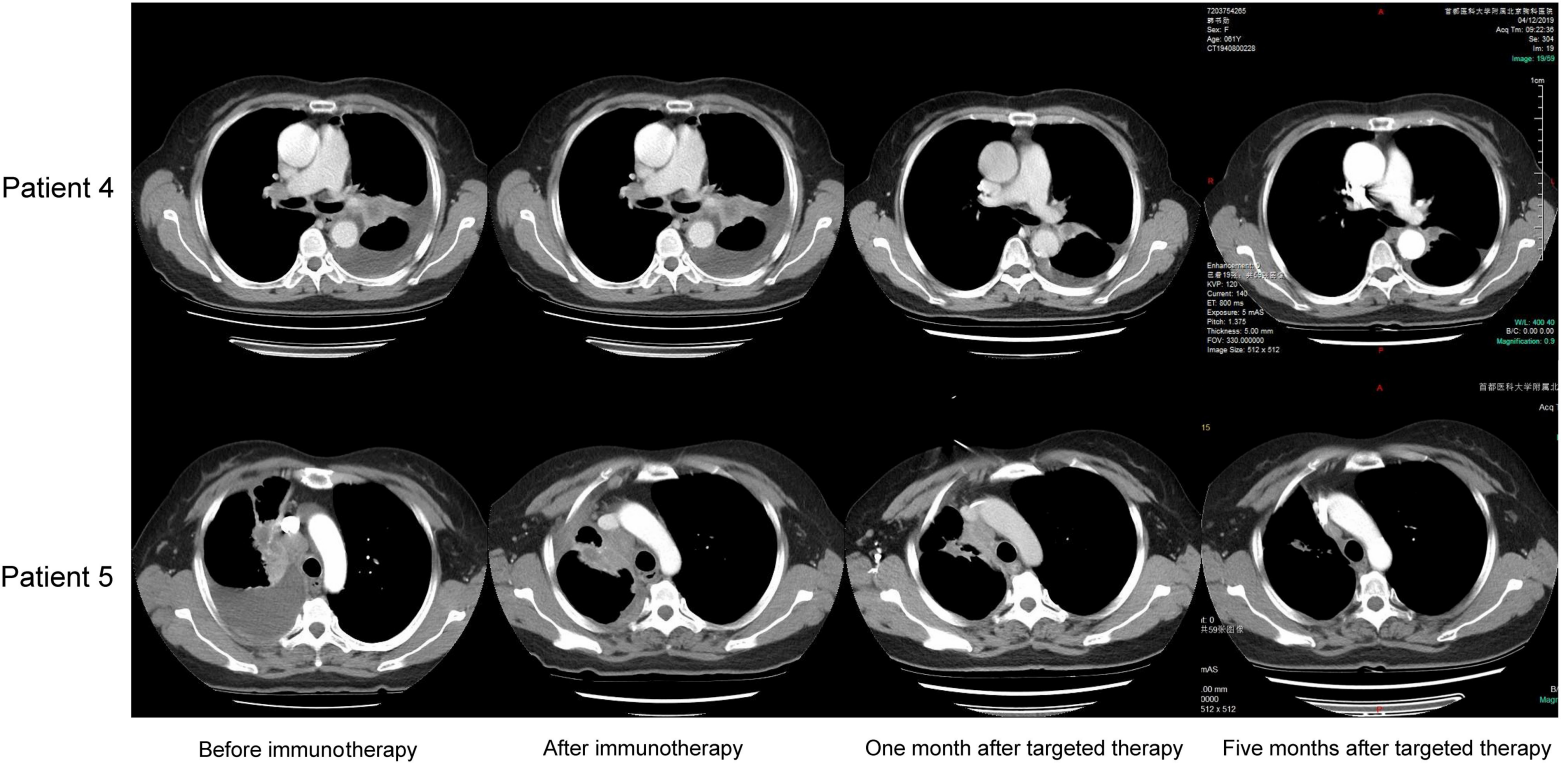
Representative images from chest CT.

Patient 4 was a 65 year-old female never-smoker who was diagnosed with advanced lung adenocarcinoma metastatic to lung, bone, mediastinal, and supraclavicular lymph nodes by checks and biopsy of the supraclavicular lymph node in April 2018. The patient received pemetrexed plus carboplatin chemotherapy as the first-line treatment. Two cycles later, she progressed with enlargement of the primary tumor, new onset of nodules in the left lung and malignant pleural effusion. Then she received nivolumab immunotherapy as the second-line treatment and confirmed progressive disease (PD) after eight cycles with new onset of bone and brain metastases. The result of NGS of re-biopsy tissue revealed HER2 exon 20 insertion mutation (p.G778_S779insCPG) (Figures 1A,C). Then, the patient received pyrotinib plus bevacizumab as the third-line therapy, together with intensity modulation radiation therapy (IMRT) for bone metastasis and EDGE radiosurgery for brain metastases. One month later, she achieved partial response (PR): The disease in lungs, mediastinal, and supraclavicular lymph nodes shrank, and pleural effusion reduced (Figure 2). The diseases in the brain also reduced, which may be a response to radiotherapy rather than targeted therapy. During the pyrotinib treatment, grade 2 diarrhea occurred, which led to temporary pyrotinib discontinuance. Four months later, the patient still kept in good performance and PFS was 12.8 months until November 30, 2019. Pyrotinib treatment continued.

Patient 5 was a 60 year-old female initially presenting with advanced lung adenocarcinoma by percutaneous lung puncture and PETCT with metastases to both lungs and mediastinal lymph nodes. She received two cycles of gemcitabine plus cisplatin and eight cycles of nivolumab with a confirmed PD. The result of NGS revealed HER2 insertion mutation (p.G780_P781dupGSP) (Figures 1B,D). Then the patient took pyrotinib as the third-line therapy with the best response of PR, and PFS was 12.5 months until November 30, 2019 (Figure 2). Grade 1 diarrhea and grade 2 nausea occurred during the treatment. The dosage was reduced for a short time and then restored the initial dose after diarrhea and nausea disappeared. Pyrotinib treatment continued. The treatment processes were summarized in Table 2.

**Table 2:** Treatment and response in patents with HER2 exon 20 insertion mutation.

**Patient**	**Mutation**	**First-line treatment**		**Second-line treatment**		**Third-line treatment**	
		**Treatment**	**Best response**	**Treatment**	**Best response**	**Treatment**	**Best response**
4	p.G778_S779insCPG	Gem+Cis	PD	Nivolumab	PD	Pyrotinib	PR
5	p.G780_P781dupGSP	Pem+Car	PD	Nivolumab	PD	Pyrotinib	PR

*Gem, gemcitabine; Cis, cisplatin; Pem, pemetrexed; Car, Carboplatin. PD, progressive disease; SD, stable disease; PR, partial response*.

Unfortunately, the other three patients did not follow the medical advice. Patients 1 and 3 still chose chemotherapy in the follow-up treatment after the NGS test, and the treatment effect was not satisfactory. Patient 2 gave up treatment because of the huge cost. Their choices should be respected, and this will not be described too much in subsequent analysis.

## Discussion

HER2 mutations represent a small subset of NSCLC. HER2 mutations were significantly associated with female, never-smoker status; advanced stage; and adenocarcinoma (19). In this study, the incidence of HER2 exon 20 insertion mutations was 1.7%. All of the patients were stage IV adenocarcinomas, which was similar to prior series of HER2-mutant NSCLCs.

The most frequent HER2 mutations described to date are insertions within a small stretch of exon 20 with A775_G776insYVMA insertion/duplication on the COOH-terminal side of the C-helix. Eng et al. (20) reports an in-frame insertion in exon 20 identified in 34 patients: 24 with identical 12 base pair insertion YVMA, 4 with 9 base pair insertions, 1 with a 6 base pair insertion, and 5 with 3 base pair insertions. A single base pair substitution was identified in four patients and included L755F, V777L, D769H in exon 20, and S310F in exon 8. Wang et al. (21) reports that 24 patients had the variants of HER2 mutation including 14 with exon20 A775_G776insYVMA, 3 with P780_Y781insGSP, 3 with G776 > VC, 2 with G776 > IC, 1 with G776 > LC, and 1 with G776C.

In this study, the HER2 exon 20 insertion mutations were scattered (shown in Table 1). Two patients had HER2 exon 20 insertions with the most common YVMA mutation. Notably, one patient had a rare HER2 exon 20 insertion mutation of G778_S779insCPG. Moreover, we found that HER2 mutations and other driver gene mutations may not be completely mutually exclusive, which was consistent with some recent research (22). The reason might be the widespread use of NGS. In addition, HER2 amplifications were common concomitant variants with HER2 mutations, and the detection rates from different studies varied widely, ranging from 8 to 57% (8, 23, 24).

According to guidelines for NSCLC, chemotherapy is still the standard therapy for patients with HER2-mutant advanced NSCLC and particularly pemetrexed-containing regimens, which are most active against lung adenocarcinomas (25). Check point inhibitors, nivolumab and pembrolizumab, are recommended as a second line for advanced or metastatic NSCLC. Mazieres et al. (22) report the RR and the median PFS for patients receiving first-line chemotherapy were 43.5% and 6 months (95% CI: 5; 7.1), respectively (*n* = 93). Most patients had first-line chemotherapy with a platinum-based doublet (without HER2-tageting treatment) (*n* = 71). In those receiving second-line chemotherapies, RR and PFS were 10% and 4.3 months, respectively (*n* = 52). Unfortunately, the patients in our study did not get any benefit from platinum-based doublet chemotherapies. Li et al. (26) reported that none of the seven patients with HER2 mutant NSCLC achieved a response to prior PD-1 inhibitors, which was similar to our patients.

Targeted therapies are highlighted in this small cohort of patients with HER2 mutations. For trastuzumab in combination with chemotherapy, RR, disease control rate (DCR), and PFS were 50, 75%, and 5.1 months, respectively (22). Lai et al. (27) report three out of 23 patients with HER2-mutant NSCLC (13%) had PR after afatinib treatment. Median duration of response of afatinib was 6 months, and median overall survival (OS) was 23 months. In a phase II study, pyrotinib, an irreversible pan-HER receptor TKI with activity against EGFR/HER1 and HER2, showed activity against HER2 mutant NSCLC and resulted in an ORR of 53.3% and a median PFS of 6.4 months (28). Except for G776>IC, patients with A775_G776insYVMA, P780_Y781insGSP, G776C, G776>VC, and L755P all responded to pyrotinib. We found not only P780_Y781insGSP, but also the rare mutation G778_S779insCPG were sensitive points for the treatment. The patients might derive more clinical benefit if they were treated at an earlier point of the disease course with a better performance status. It should be recommended to support further clinical testing of pyrotinib in NSCLC patients with HER2 exon 20 mutations to determine its impact on the OS, RR, DOR as well as the safety and toxicity.

The limitation of the paper is its retrospective design and the small sample. It is very difficult to study these less common but complex molecular subgroups of cancers. More efficient, well-designed trials, such as basket trials, might be conducted for these cancers.

In this study, HER2 exon 20 insertion mutations were found in 5 (1.7%) of the lung adenocarcinomas with a rare subtype G778_S779insCPG identified and sensitive to pyrotinib. Due to different mutation subtypes responding differently to HER2-targeted agents, the subtype should be adequately defined.

## Data Availability Statement

All data generated or analyzed during the current study are available from the corresponding author on reasonable request.

## Ethics Statement

The studies involving human participants were reviewed and approved by Institutional review committee of Beijing Chest Hospital, Capital Medical University. The patients/participants provided their written informed consent to participate in this study. Written informed consent was obtained from the patients for the publication of this case series and any potentially identifying information and images.

## Author Contributions

XZ, JL, and YW contributed to the conception, analysis, and wrote the article. NQ, LM, XL, JN, HZ, QZ, and XY provided conceptual advice, software and analysis, performed the data curation, resources, and review. HS, JW, and SZ contributed to the conception, analysis, resources, revised the original draft, and gave final approval of the version. All authors contributed to the article and approved the submitted version.

## Conflict of Interest

The authors declare that the research was conducted in the absence of any commercial or financial relationships that could be construed as a potential conflict of interest.

## Abbreviations

NSCLC: non-small cell lung cancer
HER2: Human epidermal growth factor receptor 2
NGS: next-generation sequencing
EGFR: epidermal growth factor receptor
ALK: anaplastic lymphoma kinase
RR: response rate
PFS: progression free survival
PR: partial response
PS: performance status
PD: progressive disease
OS: overall survival.
