# Supplementary material for: HER2 Exon 20 Insertion Mutations in Lung Adenocarcinoma: Case Series and Response to Pyrotinib
Source: Front Oncol. 2020 Jul 31;10:1162. doi: 10.3389/fonc.2020.01162 (PMC7411254; doi:10.3389/fonc.2020.01162)
Supplement: Supplementary file 1 [file Table_1.docx]

**Supplemental Data:**

**Summary of Previous Reports on HER2 Mutations in Lung Cancer**

| Authors | Types of HER2 mutations | Coexist mutations | Frequency | References |
| --- | --- | --- | --- | --- |
| Wang,Y., et al | Insertion  A775_G776insYVMA,  P780_Y781insGSP,  G776 > VC,  G776 > IC,  G776 > LC,  Point mutation  G776C | no | 5.1%, 25/1714 (NSCLC) | BMC Cancer, 2018[[21](#_ENREF_21)] |
| Arcila,M.E., et al | Insertion  A775_G776insYVMA  G776>VC  V777_G778insCG  P780_7781insGSP  Point mutation  L755S 1  G776C 1(concurrently with V777_G778insCG) |  | 5%, 25/560 (Adenocarcinoma)  5%, 26/507 (Adenocarcinoma)negative for major and minor EGFR and KRAS | Clin Cancer Res, 2012[[7](#_ENREF_7)] |
| Wang,Y., et al | Insertion  A775_G776insYVMA,  P780_Y781insGSP,  G776>IC,  G776>VC,  Point mutation  L755P 1PR,  G776C 1PR | no |  | Ann Oncol, 2019[[18](#_ENREF_18)] |
| Liu,Z., et al | Y772_A775dup, A775_G776insSVMA, A775_G776insVVMA, A775_G776insYVMS, Y772_V773insM-MAY,  G778_P780dup 18, 2SD(10.5%),  A775_G776insC 1 (0.6%)  G776delinsVC 14, 1PR,1SD,1PD (8.2%)  G776delinsLC 8, 1SD(4.7%) G776delinsVV 2, 1PD(1.2%) G776delinsAVGC 2 (1.2%)  G776delinsIC 3, 1PD(1.6%) G776_V777delinsCVC 2 (1.2%) | ERBB2 amplication, 20/171  (11.7%) | 2.27%, 171/7520 (lung cancer) | Onco Targets Ther, 2018[9] |
| Kris,M. G., et al | A775_G776insYVMA(50%);  P780_Y781insGSP (7.7%);  G776delinsVC (7.7%);  M774delinsWLV (3.8%);  G776delinsLC (3.8%);  V777L (3.8%);  Not specified (23%) |  |  | Ann Oncol, 2015[[14](#_ENREF_14)] |
| Eng, J., et al | Insertion  A775_G776insYVMA(70%);  G778_P780dup (12%);  G776>VC (3%);  A775_G776insC (15%);  Point mutation  L755F;  V777L;  D769H;  S310F |  |  | Lung Cancer, 2016[[20](#_ENREF_20)] |
| Lai, W.V., et al | A775_G776insYVMA15 (54%);  A775_G776insAVMA1(4);  A775_G776insVAG;  P780_Y781insGSP 1(4%);  G776>VC1(4%);  Not specified1 (4%);  D769H1 (4%);  L755F1 (4%);  V659E1 (4%);  S310F2 (7%);  lle655Val 1 (4%) |  |  | Eur J Cancer, 2019[[27](#_ENREF_27)] |
| Costa, D.B., et al | A775_G776insYVMA;  V747_G748insGSP; E740_A741insAYVM |  |  | J Thorac Oncol, 2016[[28](#_ENREF_28)] |
| Li, B.T., et al | A775_G776insYVMA;  Exon 20 insertion (9bp)  Not specified |  | 3%, 4/148 (Adenocarcinoma) | J Thorac Oncol, 2016[[29](#_ENREF_29)] |
| Stephens, P., et al | 774ins/dupins AYVM;  779ins VGS;  TT2263-4CC L755P |  |  | Nature, 2004[[30](#_ENREF_30)] |
| Mazieres, J.,et al | G776_777insVC |  | 1.7%, 65/3800 | J Clin Oncol, 2013[[8](#_ENREF_8)] |
| Kris, M.G., et al | HER2_ins.A775 |  | 3%, 19/733 (Adenocarcinoma) | JAMA, 2014[[31](#_ENREF_31)] |
| Yoshizawa, A., et al | HER2 exon 20 insertion mutations(n=161) | EGFR(n=5);  ALK (n=1); ROS-1 (n=1) |  | Ann Oncol, 2016[[23](#_ENREF_23)] |
| Suzuki,M., et al | 776_779insYVMA (87%);  G776insVC (8.8%); 775_778insAYVM (2.2%) 781_783insGSP (2.2%). | HER2 amplification, 25/44, 56.8% | 4.3%, 46/1055 (Adenocarcinoma) | Lung Cancer, 2015[[24](#_ENREF_24)] |
| Yoshizawa, A.,et al | HER2 exon 20 insertion mutations(n=6) | HER2 amplification, 2/6, 33.3% | 2.5%, 6/243 (Adenocarcinoma) | Lung Cancer, 2014[[23](#_ENREF_23)] |
| Kosaka, T., et al | A775_G776insYVMA33 G776delinsVC5; P780_Y781insGSP5; M774delinsWLV1; A775_G776insSVMA1; A775_G776insI 1;  G776delinsLC1; G778_S779InsCPG 1 ; |  | 2.5%,48/1901 | Cancer Res, 2017[[32](#_ENREF_32)] |

**Reference**

29 Costa, D. B., Jorge, S. E., Moran, J. P., Freed, J. A., Zerillo, J. A., Huberman, M. S. *et al.* Pulse Afatinib for ERBB2 Exon 20 Insertion-Mutated Lung Adenocarcinomas. *Journal of thoracic oncology : official publication of the International Association for the Study of Lung Cancer*. (2016) 11: 918-923. doi:10.1016/j.jtho.2016.02.016

30 Li, B. T., Ross, D. S., Aisner, D. L., Chaft, J. E., Hsu, M., Kako, S. L. *et al.* HER2 Amplification and HER2 Mutation Are Distinct Molecular Targets in Lung Cancers. *Journal of thoracic oncology : official publication of the International Association for the Study of Lung Cancer*. (2016) 11: 414-419. doi:10.1016/j.jtho.2015.10.025

31 Stephens, P., Hunter, C., Bignell, G., Edkins, S., Davies, H., Teague, J. *et al.* Lung cancer: intragenic ERBB2 kinase mutations in tumours. *Nature*. (2004) 431: 525-526. doi:10.1038/431525b

32 Kris, M. G., Johnson, B. E., Berry, L. D., Kwiatkowski, D. J., Iafrate, A. J., Wistuba, II *et al.* Using multiplexed assays of oncogenic drivers in lung cancers to select targeted drugs. *Jama*. (2014) 311: 1998-2006. doi:10.1001/jama.2014.3741

33 Kosaka, T., Tanizaki, J., Paranal, R. M., Endoh, H., Lydon, C., Capelletti, M. *et al.* Response Heterogeneity of EGFR and HER2 Exon 20 Insertions to Covalent EGFR and HER2 Inhibitors. *Cancer Res*. (2017) 77: 2712-2721. doi:10.1158/0008-5472.CAN-16-3404
